# Effects of Lip Color on Perceived Lightness of Human Facial Skin

**DOI:** 10.1177/2041669517717500

**Published:** 2017-07-11

**Authors:** Yuki Kobayashi, Soyogu Matsushita, Kazunori Morikawa

**Affiliations:** School of Human Sciences, Osaka University, Japan; Faculty of Liberal Arts, Osaka Shoin Women’s University, Japan; School of Human Sciences, Osaka University, Japan

**Keywords:** assimilation, cosmetics, face, illusion, lightness, lip

## Abstract

Whereas geometric illusions in human faces have been reported by several studies, illusions of color or lightness in faces have seldom been explored. Here, we psychophysically investigated whether lip color influences facial skin’s perceived lightness. Results of Experiment 1 demonstrated that redder lips lightened and darker lips darkened the perceived complexion. These lightness or darkness inducing effects differ from the classical illusion of lightness contrast in nonface objects for two reasons. First, illusory effects are more assimilative than contrastive. Second, the inducing area (i.e., lips) is much smaller than the influenced area (facial skin). Experiment 2 showed that the assimilative lightness induction was caused by holistic processing of faces. This is the first study to scientifically substantiate the claim of cosmetics manufacturers and makeup artists that lip colors can alter perceived facial skin color. Implications for face perception, lightness illusion, and perceptual effects of cosmetics are discussed.

## Introduction

This study examined interaction among three fields of perceptual study: face perception, lightness illusion, and perceptual effects of cosmetics. Face perception and lightness illusion (e.g., lightness perception is affected by the lightness of adjacent areas) have been investigated separately by numerous studies (Adelson, 2000; Tanaka & Farah, 1993) and are already established as different research fields with very little relationship. In addition, in recent years, perceptual effects of cosmetics have begun to draw attention as a promising new field of perceptual study (Matsushita, Morikawa, Mitsuzane, & Yamanami, 2015; Morikawa, Matsushita, Tomita, & Yamanami, 2015). However, very few studies, if any, have tackled the intersection of these three fields.

Although several studies have examined shape or size illusions in faces (Lee & Freire, 1999; Matsushita, Morikawa, & Yamanami, 2015; Morikawa et al., 2015), illusion of facial color or lightness remains largely unexplored. Makeup artists and advertisements for cosmetics often claim that lip color can influence facial skin’s apparent lightness (e.g., http://verilymag.com/2015/02/best-lipstick-skin-tone). Currently, we do not have scientific evidence to either support or deny these claims.

Previous studies have revealed that face color or lightness is a major determinant of perceived healthiness and attractiveness (Frost, 1990; Pazda, Thorstenson, Elliot, & Perrett, 2016; Stephen, Coetzee, & Perrett, 2011). Moreover, contrast between light facial skin and dark facial parts (eyes and lips) is positively correlated with perceived health, femininity, and attractiveness (Jones, Russell, & Ward, 2015; Russell, 2003, 2010). In fact, one way for cosmetics to make the face appear more feminine and, hence, attractive is by exaggerating facial contrast (Jones et al., 2015; Russell, 2010). Although cosmetics’ effect on attractiveness may not be large (Jones & Kramer, 2015, 2016), color and lightness of lips can be easily and dramatically altered using lipsticks. Lipsticks may not only increase the contrast between lips and facial skin but may also influence perceived lightness of skin. Therefore, it would be both scientifically interesting and beneficial for practical purposes to test the effect of lip color on facial skin’s perceived lightness.

On one hand, there is a reason for thinking that lip color would not affect facial skin’s perceived lightness: The lip area is far smaller than the facial skin area. Generally, in nonface objects and surfaces, color or lightness of a larger and surrounding area influences perceived color or lightness of a smaller and surrounded area but not vice versa (Yund & Armington, 1974). Although the smaller area might affect the larger surrounding one, it would be too weak to observe. Lips are much smaller than the facial skin that surrounds them. Considering these facts, we might predict that lip color or lightness is unlikely to affect that of the larger and surrounding area, that is, facial skin.

On the other hand, many researchers have argued that processing faces differs from processing nonface objects because the former relies more heavily on holistic processing than does the latter (Cornes, Donnelly, Godwin, & Wenger, 2011; Rossion, 2013; Tanaka & Farah, 1993). Therefore, the lightness-induction pattern in faces can possibly differ from that in nonface objects. Morikawa (2017) noted that geometric illusions in human faces and bodies tend to occur in the direction of assimilation rather than contrast. Morikawa argued that this pattern of geometric illusions is distinct from classical nonface illusions, calling it “biological illusion.” If this peculiar tendency occurs in lightness perception of faces, lip color might affect perceived facial skin tone. Investigating lightness illusion in human faces and comparing it with the illusion in nonface objects could contribute to studies of both lightness perception and face perception. Therefore, the present study attempted to examine whether lip color and lightness could affect perceived lightness of facial skin.

## Experiment 1

### Method

This study was approved by the Research Ethics Committee of the School of Human Sciences, Osaka University. Twenty-one naïve volunteers (10 females) participated in Experiment 1. Their ages ranged from 20 to 32 years (*M* = 23.4, *SD* = 2.54). They had normal or corrected-to-normal visual acuity and normal trichromatic color vision and gave written informed consent. The sample size of this study is much larger compared with that of typical lightness perception studies (e.g., Madigan & Brainard, 2014; Sawayama & Kimura, 2012). Stimuli were presented on an LCD screen (NEC MultiSync LCD-PA241W, screen size 518 × 324 mm, 1920 × 1200 pixels, refresh frequency 60 Hz, CIE chromaticity coordinates of white: *x* = 0.322, *y* = 0.326) by a computer program created with PsychoPy2 1. 82. 01 (Peirce, 2007, 2008). The viewing distance was secured at 57 cm by using a chin rest. The room was normally illuminated.

All facial stimuli were generated from an average face of 40 Japanese women in their 20s. They were asked to keep a neutral expression while being photographed under the same diffused illumination. Software Morph 2.5 J was used to average those faces. Dimensions of stimulus images were 816 pixels wide and 1052 pixels high (220 mm wide and 284 mm high), including the white background (113.8 cd/m^2^) of the faces. The present study is one of the very first attempts at measuring color or lightness illusion caused by lip color; therefore, we adopted an average face as a starting point.

**Figure 1.**
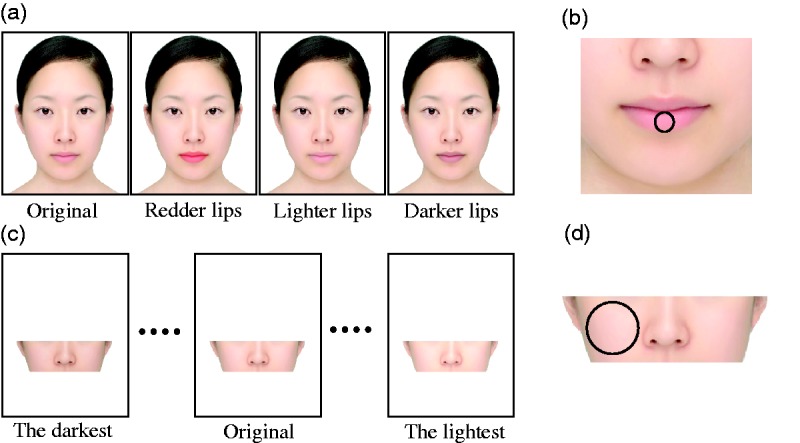
(a) Four standard stimuli. They were identical faces except for lip colors. (b) The area where luminances and chromaticity coordinates were measured is indicated by a circle on the lower lip. (c) Examples of comparative stimuli. Nine levels were prepared. (d) The area where luminances were measured in each comparative stimulus is indicated by a circle on the cheek.

Four standard stimuli were employed (Figure 1(a)). One was the original face, and the others were faces with lighter lips, darker lips, and redder lips. Because our main interest was lightness illusion, the standard stimuli included darker and lighter lips. A previous study reported that reddish facial skin is perceived lighter (Yoshikawa, Kikuchi, Yaguchi, Mizokami, & Takata, 2012). If lip color is assimilated with facial skin color, then redder lips might make facial skin appear lighter. Therefore, we also included redder lips in the standard stimuli. Adobe Photoshop CS3 was used to create these altered lips. Using the Hue or Saturation function, we decreased the lightness value by 8 for darkening and we increased it by 12 for lightening. Using the Color Balance function, we increased the red value by 100 for reddening. Dimensions of the face itself were approximately 544 pixels (147 mm) wide at eye level and approximately 937 pixels (253 mm) high from the top of the head to the tip of the chin. The average luminance and CIE chromaticity coordinates of a small circular area covering the lower lip (Figure 1(b)) were as follows, respectively: original: 53.5 cd/m^2^, (*x*, *y*) = (0.414, 0.347); redder lips: 45.0 cd/m^2^, (*x*, *y*) = (0.466, 0.340); lighter lips: 56.0 cd/m^2^, (*x*, *y*) = (0.397, 0.353); and darker lips: 43.7 cd/m^2^, (*x*, *y*) = (0.414, 0.347). The darker lips’ luminance was lowered by more than the lighter lips’ luminance was raised because lipsticks which darken the lips are much more common than the ones which lighten them for everyday makeup (Russell, 2009).

Comparative stimuli consisted only of the middle slice of the average face for three reasons (Figure 1(c)). First, the part of the human face that represents “complexion” the best is cheeks and not the forehead or chin. Second, it is well known that lightness contrast or assimilation illusions are sensitive to the spatial separation between the inducing area and the test area. If lip color has any illusory effect at all, it would most likely manifest itself in skin areas near the lips and not in the forehead. Third, we wanted participants to compare luminance in the same part of the face. If the comparative stimuli had consisted of an entire face, some participants may have compared the forehead while other participants may have compared the nose, cheeks, and so on. Nine levels of luminance were prepared: 60.5, 63.0, 65.4, 67.6, 69.9, 71.9, 73.9, 75.7, and 77.5 cd/m^2^. The luminance of each stimulus indicates the average of a circular area covering the cheek (Figure 1(d)).

The method of constant stimuli was employed. In a trial, one standard stimulus and one comparative stimulus were presented side by side on a gray background (56.4 cd/m^2^). The distance between the two stimuli was 144 pixels (39 mm), and they were aligned so that the partial face was exactly level with the corresponding part in the full face. Participants were asked to report which facial skin appeared lighter by pressing a key. The stimuli presentation lasted at most for 1,500 ms, but it was interrupted by participants’ key presses. Between trials, there were 500 ms intervals during which only the gray background was displayed. Experiment 1 was composed of 288 trials (four standard stimuli, nine comparative stimuli, two presentation positions, and four repeats), and trial order was randomized. Participants began the experiment after a practice of 40 trials (four standard stimuli, five comparative stimuli, and two presentation positions).

### Results

**Figure 2.**
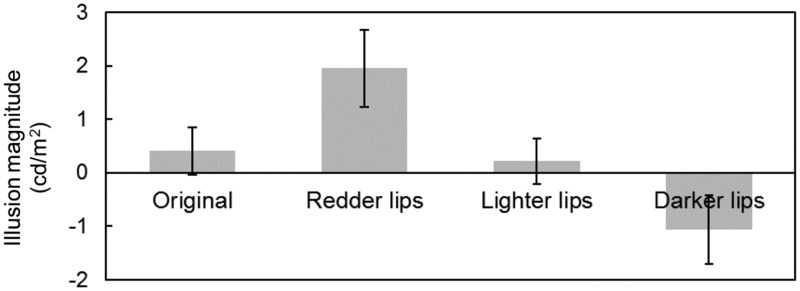
Illusion magnitude of perceived luminance for each standard stimulus in Experiment 1. Error bars indicate standard errors.

We obtained psychometric functions from luminance of each comparative stimulus and its selection probability for each participant. Then, a cumulative normal distribution function was fitted to individual data by the least squares method. The luminance value (the horizontal axis) of the function when the probability value (the vertical axis) reached 50% (i.e., the mean) was defined as the point of subjective equality (PSE), where perceived lightness of the standard stimulus was equal to that of the comparative stimulus. To properly fit the function to individual data, it was expected that the selection probability for the darkest and lightest comparative stimuli should be 0% and 100%, respectively (these two extreme comparative stimuli were created as obviously darker or lighter than the standard stimulus). For these two stimuli, we set a criterion of 75% correct responses, which is a common definition of threshold employed in two-alternative forced-choice psychophysical experiments. One male participant fell short of the criterion. Therefore, he was excluded, and the remaining 20 were further analyzed. Figure 2 shows the mean of PSE for each standard stimulus. A one-way repeated measures analysis of variance indicated that the main effect of lip color was significant, *F*(3, 57) = 15.07, *p* < .001, η_p_^2 ^= .44. Post hoc *t* tests using Holm’s method (Holm, 1979) was performed on the data. Holm’s method, which employs sequentially rejective tests, is known for its versatility as well as Bonferroni’s method; however, its Type II error rate is lower than Bonferroni’s method. The following *p* values were sequentially corrected with Holm’s method. *T* tests showed that the facial skin with redder lips appeared lighter than that of the original face, *t*(19) = 2.94, *p* = .017, *d* = 0.57, and the facial skin with darker lips appeared darker than that of the original face, *t*(19) = 4.03, *p* = .004, *d* = 0.58. However, there was no significant difference between the skin with lighter lips and the original face, *t*(19) = 1.18, *p* = 1.00, *d* = 0.10. Regarding sex differences, neither main effect of sex nor interaction between sex and lip color was significant (*p* = .066 and *p* = .932, respectively).

These results revealed that redder lips lightened perceived lightness of facial skin and that darker lips darkened it. In other words, lip color can influence facial skin’s appearance. Here, we could not observe lighter lips’ effect but that might be because the luminance increment for the lighter lips was relatively small. Results of Experiment 1 showed that lip color can cause lightness assimilation in human faces. The effect of redder lips will be discussed in the Discussion section.

Normally, as mentioned earlier, lightness induction from a small and surrounded area to a large and surrounding area is unlikely to arise. Possibly, this unusual pattern of lightness induction is caused by holistic processing, which is known as a peculiarity of human face processing that can be disrupted by inversion (i.e., turning upside down; e.g., Tanaka & Farah, 1993; Thompson, 1980; Valentine, 1988). Therefore, we conducted a face inversion experiment to examine whether this effect can be explained by holistic processing. If the lightness-inducing effects observed in Experiment 1 were caused by holistic processing, they should be much weaker in Experiment 2.

## Experiment 2

### Method

Twenty-two volunteers (12 females) participated in Experiment 2; their ages ranged from 19 to 44 years (*M* = 24.6, *SD* = 4.79). They had normal or corrected-to-normal visual acuity and normal trichromatic color vision and gave written informed consent. None of them participated in Experiment 1.

The apparatus and procedure were the same as in Experiment 1 except that all the stimuli were inverted.

### Results

**Figure 3.**
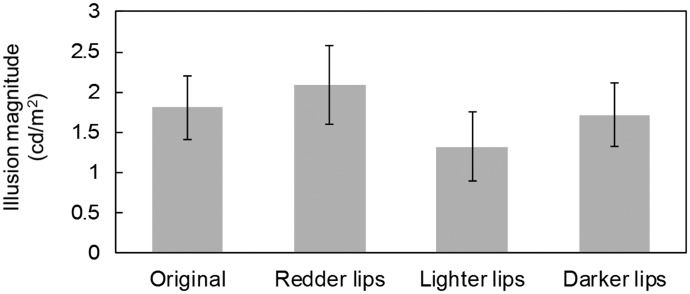
Illusion magnitude of perceived luminance for each standard stimulus in Experiment 2. Error bars indicate standard errors.

Two female participants were excluded from analysis by the aforementioned criterion, and the remaining 20 were analyzed. PSEs were calculated using the same method as in Experiment 1. Figure 3 shows consistently high PSEs, which may be because the inverted comparative stimuli were more vulnerable to disruption of holistic processing. If the inverted partial face is not perceived as a face, facial lightness constancy fails, which makes the partial face subject to simultaneous contrast effect and perceptually darkened by the surrounding white area (Yund & Armington, 1974). A one-way repeated measures analysis of variance showed that the main effect of lip color was not significant, *F*(3, 57) = 2.43, *p* = .074, η_p_^2 ^= .11. Post hoc *t* tests using Holm’s method (Holm, 1979) showed no significant differences between the original face and the other three conditions (original vs. redder lips: *t*(19) = 0.98, *p* = .680, *d* = 0.14; original vs. lighter lips: *t*(19) = 1.66, *p* = .565, *d* = 0.26; original vs. darker lips: *t*(19) = 0.466, *p* = .647, *d* = 0.05). Regarding sex differences, neither main effect of sex nor interaction between sex and lip color was significant (*p* = .755 and *p* = .766, respectively).

Unlike Experiment 1, the lightness-inducing effects could not be observed in Experiment 2. This shows that the lightness-inducing effects of lips on facial skin stem largely from holistic processing.

## Discussion

Experiment 1 revealed that redder lips made perceived lightness of facial skin lighter and that darker lips made it darker. Previous studies with nonface stimuli (Yund & Armington, 1974) showed that a smaller and surrounded area is unlikely to cause lightness induction in a larger surrounding area. Therefore, these lightness-inducing effects observed in Experiment 1 may be unique to faces. Results of Experiment 2 supported this hypothesis, showing that such effects cannot be observed in inverted face stimuli. It is likely that holistic processing of faces causes assimilative lightness induction. Below, we discuss implications of the present findings in three fields of perceptual research: face perception, lightness illusions, and effects of facial makeup.

Morikawa’s (2017) “biological illusion,” mentioned earlier, could explain this unique pattern of lightness induction. According to his argument, illusions in human faces and bodies share tendencies uncommon in nonface objects. One tendency is assimilative: Geometric illusions in body parts tend to induce illusions of assimilation instead of contrast. And Morikawa also observed that in the human face and body, the visual property of a smaller region can spread to larger adjacent areas, a phenomenon that he called “visual echo illusion.” These tendencies of biological illusion have been confirmed by some other studies (Matsushita et al., 2015; Morikawa, 2017). Indeed, the present study’s results seem to accord with the theory of biological illusion.

Although at first glance the phenomenon that redder lips lightened perceived lightness of skin does not appear to accord with tendencies of biological illusion, the phenomenon can be reconciled with biological illusion if we consider the finding that reddish faces appear lighter (Yoshikawa et al., 2012). More specifically, facial skin’s reddish appearance caused by redder lips’ assimilative illusion would lead to lighter appearance of complexion. Therefore, effects of both darker lips and redder lips can be considered illusions of color or lightness assimilation in accord with characteristics of biological illusion.

Kiritani, Okazaki, Motoyoshi, Takano, and Okubo (in press) also reported that lip color was assimilated into facial skin’s perceived color. For example, they found that orange lip color makes facial skin appear yellowish, and reddish lip color makes facial skin appear reddish and lighter. Their findings are in general agreement with ours. However, Kiritani et al.’s (in press) experiment employed paired comparisons instead of psychophysical methods, so the absolute size of illusion was unclear. In addition, in their experiment, both standard stimuli and comparative stimuli were whole faces including lips, and participants were asked to evaluate colors of “whole faces.” Possibly, therefore, participants judged the average color of the whole face including lips. That is, their results might have derived not only from judgment of facial skin color but also from that of lip color itself. The present study overcame those problems by using a psychophysical method and partial faces excluding lips as comparative stimuli.

To the best of our knowledge, this study presents the first psychophysical evidence that supports the claim of cosmetics manufacturers and makeup artists that lipsticks can influence perceived lightness of facial skin. Lip color has been known as a major determinant of facial attractiveness and sex typicality (Stephen & McKeegan, 2010). Lipsticks can directly increase contrast between lips and facial skin, which leads to increased perceived health, femininity, and attractiveness (Jones et al., 2015; Russell, 2003, 2010). In addition, we discovered the existence of an indirect lip-color effect on the impression of faces mediated by the illusion of facial skin’s perceived lightness. Facial skin lightness is related to sexual dimorphism and apparent healthiness (e.g., Frost, 1990; Stephen et al., 2011). Moreover, the illusory effect of red lip-color on facial skin can enhance facial contrast, which increases perceived femininity, health, and youthfulness (Porcheron, Mauger, & Russell, 2013; Russell et al., 2016). Therefore, cosmetics have both direct and indirect illusory effects to enhance facial beauty, femininity, and attractiveness.

Still, some unresolved issues remain to be investigated by future studies. First, a serious weakness of the present study is the fact that it used only one average face. Therefore, the generalizability of our conclusions is limited. Follow-up studies that employ a wide variety of individual faces are needed to examine whether the lightness assimilation also occurs with other faces. Second, effects of lightness and saturation of lips’ redness would have to be disentangled. Third, we do not yet understand how the lightness assimilation spreads from the lips. For example, it may be possible that darkening the lips makes the immediate area around the lips appear lighter, but areas further away (the cheeks) appear darker. Finally, the effect of lighter lips was not observed, but it might be because of the small increment in the lips’ luminance. Much lighter lips might have increased the perceived lightness of skin significantly. Further research is necessary to investigate the exact nature of the facial lightness illusion.

The present study demonstrated that unusual illusions of lightness or color assimilation can arise in face perception because of holistic processing of faces. In addition, it was suggested that psychophysical methods can be very useful for measuring perceptual effects of cosmetic facial makeup. Finally, it was proposed that faces should be studied as unique objects worthy of academic attention from the perspective of lightness perception. The present study could make new contributions to three fields of perceptual research: face perception, lightness illusion, and visual effects of cosmetics.
