# Corrigendum

**DOI:** 10.1177/2041669519865375

**Published:** 2019-08-08

Kobayashi, Y., Matsushita, S. & Morikawa, K. (2017). Effects of Lip Color on Perceived Lightness of Human Facial Skin. *i-Perception, 8*(4): 1–10. doi: 10.1177/2041669517717500

In this study, there was an error in processing data from one participant in Experiment 2 on page 5. Correcting the data resulted in slight changes in the text on page 6, line 7 under “Result” section and

**Figure 3.**
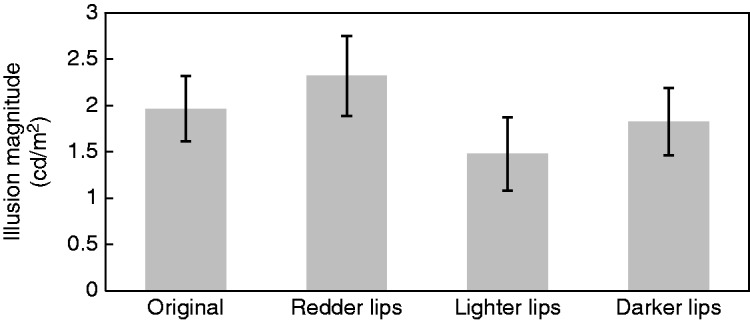
Illusion magnitude of perceived luminance for each standard stimulus in Experiment 2. Error bars indicate standard errors.

Figure 3. This corrigendum does not change the conclusion of the paper.

The updated text on page 6 and Figure 3 should read and appear as:

A one-way repeated measures analysis of variance showed that the main effect of lip color was significant, *F*(3, 57) = 3.04, *p* = .036, $\eta_{\text{p}}^{2}$ = .14. However, post hoc *t* tests using Holm’s method (Holm, 1979) showed no significant differences between the original face and the other three conditions (original vs. redder lips: *t*(19) = 1.25, *p* = .456, *d* = 0.20; original vs. lighter lips: *t*(19) = 1.68, *p* = .549, *d* = 0.29; original vs. darker lips: *t*(19) = 0.747, *p* = .464, *d* = 0.09). Regarding sex differences, neither main effect of sex nor interaction between sex and lip color was significant (*p* = .918 and *p* = .910, respectively).

Unlike Experiment 1, effects of lip colors were not observed in Experiment 2. This shows that the lightness-inducing effects of lips on facial skin stem largely from holistic processing.
